# Essential Oil from the Leaves of the Dwarf Cashew Tree (*Anacardium occidentale* L.) in the Amazon Savannah: Physicochemical and Antioxidant Properties as a Food Preservative

**DOI:** 10.3390/foods14111954

**Published:** 2025-05-30

**Authors:** Maria Clarisnete de Oliveira Moura, Esther Morais da Silva Assuncão, Salatiel Silva Barbosa, Edu Istarley Lourenço Tenente, Alessandro Pereira de Souza, Rajá Vidya Moreira dos Santos, Ana Paula Folmer Correa, Laura Adriane de Moraes Pinto, Amélia Carlos Tuler, Daniela Cavalcante dos Santos Campos, Marcos Jose Salgado Vital, Antonio Alves de Melo Filho, Jéssica de Oliveira Monteschio

**Affiliations:** 1 Bionorte Network Postgraduate Program in Biodiversity and Biotechnology, Federal University of Roraima, Boa Vista, RR 69310-000, Brazil; 2 Agrarian and Environmental Sciences Institute, Federal University of de Mato Grosso, Sinop, MT 78060-900, Brazil; 3 Department of Animal Science, Federal University of Roraima, Boa Vista, RR 69310-000, Brazil; 4 Chemistry Department, Federal University of Roraima, Boa Vista, RR 69310-000, Brazil; 5 Postgraduate Program in Natural Resources, Federal University of Roraima, Boa Vista, RR 69310-000, Brazil; 6 Department of Food Engineering, Midwest State University, Guarapuava, PR 85040-167, Brazil; 7 Biodiversity Study Center, Federal University of Roraima, Boa Vista, RR 69310-000, Brazil; 8 Agrotechnical School, Federal University of Roraima, Campus Murupu, Boa Vista, RR 69310-000, Brazil

**Keywords:** antioxidant, characterization, essential oil, food, terpinolene, yield

## Abstract

*Anacardium occidentale*, known as cashew tree, is widely used in the Amazon. This study aimed to evaluate the chemical composition, as well as the biological, physicochemical, antioxidant, and acceptability properties, of the essential oil (EO) extracted from the leaves of the dwarf cashew tree (EOLC) from the Amazonian savanna. The EO was obtained by hydrodistillation from fresh and frozen leaves, with the frozen sample selected due to its higher yield. The components of the EOLC were identified by GC-MS. Antioxidant activity was assessed using DPPH and ABTS radicals, with values of 1.96 µmol Trolox mL^−1^ and 1.41 mM, respectively. Total phenolic content was determined by the Folin–Ciocalteu method. Antibacterial activity was evaluated by agar diffusion and minimum inhibitory concentration (MIC) methods, and toxicity was assessed with *Artemia salina* L. The physicochemical properties analyzed included density, refractive index, viscosity, and solubility. Terpinolene was identified as the major compound (80.21%). The EOLC exhibited antioxidant capacity, stronger antibacterial action against Gram-positive bacteria, moderate toxicity, and appropriate physicochemical characteristics. The 0.05% concentration was the most accepted in the sensory evaluation, standing out as a promising natural alternative for application in meat products. These findings highlight the potential of EOLC as a natural food preservative and a source of bioactive compounds, with promising applications in various food matrices.

## 1. Introduction

The cashew tree (*Anacardium occidentale* L.), belonging to the Anacardiaceae family, is a tropical fruit tree widely cultivated in Brazil, especially in the Northeast and Amazon regions, where it plays a significant economic and social role [1,2,3]. Its main commercial value lies in the production of the cashew nut, the true fruit, and the pseudofruit, known as the cashew apple, both of which are widely used in the food industry. The nut, rich in essential fatty acids and bioactive compounds, is a highly nutritious product with export value [4]. The pseudofruit, in turn, is a rich source of polyphenols, vitamins, and minerals, and is consumed fresh or processed into juices, sweets, and liqueurs [5,6].

Although the fruits and pseudofruits are the most commercially exploited parts, the cashew tree leaves represent an abundant yet underutilized source of biomass. Previous studies have shown that the leaves contain relevant bioactive compounds, including flavonoids [7], tannins, alkaloids, saponins [8], terpenes [9], and essential oils [10,11], which are associated with various biological activities, such as antimicrobial, antioxidant, anti-inflammatory, and anticancer effects [12,13].

In addition to their phytochemical potential, the leaves also exhibit important therapeutic properties and are traditionally used in folk medicine to treat gastrointestinal disorders, fevers, inflammation, and diabetes [14,15]. The therapeutic relevance of this species is officially recognized, as *A. occidentale* is included in the National List of Medicinal Plants of Interest to the Brazilian Unified Health System (RENISUS), which comprises species with potential for therapeutic use in public healthcare [16].

There are two main varieties of cashew tree: the common type, which can grow over 15 m tall, and the dwarf precocious cashew tree, which is smaller, reaching up to 6 m. The latter stands out for its shorter productive cycle, flowering and fruiting as early as the first year of cultivation [17]. Among the cultivated varieties of dwarf cashew tree, the CCP 76 clone is notable for its high productivity, reduced height, and excellent fruit quality, with attractive sensory characteristics for consumers, such as greater sweetness and lower astringency [17]. These attributes favor its use both for fresh consumption and for industrial processing, making it an interesting target for new research and applications.

Brazil has one of the richest biodiversities on the planet, with the Amazon Rainforest considered the largest gene bank in the world [18,19]. In recent years, there has been great global interest in the development of new technologies that enable the use of products with a lower environmental impact, with potential use in human nutrition [19,20]. In this sense, the use of aromatic and medicinal plants, as a source of essential oils and other bioactive compounds, presents itself as a promising alternative, especially due to the availability of renewable resources and the need for environmental conservation.

Essential oils have been widely investigated for their broad spectrum of biological properties, including antioxidants, antimicrobial, antitumor, anti-inflammatory, and analgesic activities [21,22]. Chemically, they are composed of complex mixtures of low molecular weight volatile substances, such as terpenes, phenylpropanoids, and oxygenated derivatives including alcohols, aldehydes, ketones, acids, phenols, lactones, ethers, and esters [23,24]. These compounds can be extracted from different plant parts, including flowers, leaves, stems, bark, seeds, and roots [25]. In addition, essential oils are recognized for their various pharmacological effects, including antispasmodic, hepatoprotective, antiviral, and anticancer activities [26].

It is important to highlight that, to date, there are very few published studies on the physicochemical and biological properties of the essential oil extracted from the leaves of the early dwarf cashew tree, especially from plants grown in the Amazon region. This scenario lends originality to the present work, which aimed to evaluate the extraction efficiency and characterization of the essential oil from the leaves of the early dwarf cashew tree (clone CCP 76), focusing on its physicochemical, antioxidant, biological and sensory properties, aiming at its potential application in the food sector.

## 2. Materials and Methods

### 2.1. Species Collection and Identification

The leaves of the early dwarf cashew tree were collected on a property located in the municipality of Normandia, Roraima (latitude 3°53′07.5′′ N; longitude 59°40′10.9′′ W). Three individuals of the clone CCP 76, developed by the Brazilian Agricultural Research Corporation (EMBRAPA, Ceará, Brazil), recognized for presenting pseudofruits with a sweet flavor [17], were selected. The voucher specimens were deposited in the Herbarium of the Center for Biodiversity Studies (CBio) of the Federal University of Roraima (UFRR), under registration number 9975.

The leaves were packaged in polypropylene bags and transported to the Chemistry Laboratory of the Federal University of Roraima (UFRR). Part of the material was used immediately for the extraction of essential oil from fresh leaves, while another part was stored and frozen in a freezer at −20 °C for a period of 15 days, for later extraction of essential oil from frozen leaves. The extraction of essential oil from fresh and frozen leaves had as its main objective the comparison of yields between the two processing conditions.

### 2.2. Extraction and Yield of EOLC

Essential oil from the leaves of the early dwarf cashew tree was obtained by the hydrodistillation process using a Clevenger-type device with a double condenser adapted from Spell’s model. The extraction procedure was based on the official protocol of the Adolfo Lutz Institute [27], with adaptations. Initially, 2 kg of leaves were cut into small pieces and placed in a 12 L flask, with 8 L of distilled water being added, corresponding to a ratio of 1 kg of leaves for every 4 L of water. The flask was heated for an uninterrupted period of 3 h, counting from the moment of condensation of the first drop, to a temperature of approximately 60 °C. The same process for extracting the essential oil from fresh leaves of the dwarf cashew tree was carried out to extract the essential oil from frozen leaves.

After the extraction period, the EOLC were completely separated from the aqueous phase and placed in a container wrapped with aluminum foil, closed and kept in a freezer (−20 °C). The yield (%) was calculated according to Equation (1):

Essential oil yield (%) = (m_1_/m) × 100, (1)

where m_1_ is the mass in g of the distilled essential oil and m is the mass in g of the sample [27]. The extraction and yield of essential oils were performed in triplicate.

For the analysis of chemical composition, biological, physicochemical, and acceptability properties, the essential oil sample that showed the highest yield between the two evaluated conditions was used.

### 2.3. Determination of the Chemical Compounds

The chemical composition of the essential oil from cashew tree leaves was determined by gas chromatography coupled to mass spectrometry (GC-MS), using a TRACE GC ULTRA/ISQ system (Thermo Scientific, Waltham, MA, USA) equipped with a TR-5 capillary column (30 m × 0.25 mm × 0.25 μm). Helium was used as the carrier gas at a flow rate of 1.0 mL/min. The injection solution was prepared by dissolving approximately 1 mg of essential oil in 1 mL of HPLC-grade ethyl acetate. Then, 1 μL of the solution was injected in split mode at a ratio of 1:30. The oven temperature was programmed according to Silva et al. [28]. The retention index (RI) was calculated for all volatile components using a homologous series of n-alkanes (C8–C20), according to the equation of Van den Dool and Kratz [29]. The mass spectra of the identified chemical compounds were compared with those available in the NIST 20 library, and the identification was confirmed by comparing the retention indices reported in the literature [30].

### 2.4. Determination of Antibacterial Activity

#### 2.4.1. Bacterial Strains and Culture Conditions

To prepare the microorganism suspensions, five species of bacteria were used as indicators to evaluate the antibacterial activity of EOLC, which included three Gram-positive bacteria, *Staphylococcus aureus* (ATCC 1901, standard strain), *Listeria monocytogenes* (ATCC 7644, standard strain), and *Bacillus cereus* (ATCC 9634, standard strain), and two Gram-negative bacteria, *Salmonella enteritidis* (ATCC 13076, standard strain) and *Escherichia coli* (ATCC 10536, standard strain). The selected bacterial strains are widely recognized for their relevance in food safety, as they are common pathogens frequently associated with food contamination [31,32]. The choice of these species aims to reflect the efficacy of EOLC against important food pathogens, reinforcing the relevance of investigating its use as a natural preservative.

The cultures were inoculated separately in sterile BHI broth (Kasvi, Curitiba, Brazil) and incubated for 24 h at 35 °C to reach the logarithmic growth phase. Subsequently, the cells obtained were standardized at 10^8^ CFU/mL, according to the McFarland Scale [33].

#### 2.4.2. Agar Diffusion Method

The antibacterial activity was determined according to the methodology described by Meira et al. [34]. A bacterial suspension containing 10^8^ CFU/mL was prepared in sterile saline solution (0.85% *w*/*v* NaCl) was used to inoculate Mueller Hinton agar plates (Sigma-Aldrich, St. Louis, MO, USA) with the aid of a swab. Subsequently, sterile filter paper discs (6 mm in diameter) were impregnated with 15 μL of pure EOLC and positioned on the surface of the previously inoculated plates. The plates were incubated at 37 °C for 24 h to verify the formation of zones of inhibition. The diameters of the zones of growth inhibition were measured in millimeters using an antibiogram (in mm) [35]. Chloramphenicol was used as a positive control to determine bacterial sensitivity. Each essential oil sample and bacterial variant was tested in triplicate, and the inhibition zone diameters were reported as mean ± standard deviation.

#### 2.4.3. Determination of Minimum Inhibitory Concentration (MIC)

The minimum inhibitory concentration (MIC) of the EOLC was determined using the broth microdilution method, as described by Granado et al. [36]. Serial dilutions of EOLC were prepared in Mueller-Hinton medium (Sigma-Aldrich, St. Louis, MO, USA), supplemented with 1% (*v*/*v*) Tween 80 (Merck, Darmstadt, Germany) as an emulsifier, to improve the dispersion of the essential oil in the aqueous medium. The concentrations tested ranged from 0.0156 to 2.0 mg/mL.

In sterile 96-well microdilution plates, 100 µL of the standardized bacterial suspension and 100 µL of the EOLC dilution were added to each well. The solvent control (medium with Tween 80 without oil) and the negative control (medium without inoculum) were included, aiming to guarantee the reliability of the results.

The plates were incubated at 37 °C for 24 h under aerobic conditions. The reading was performed by measuring turbidity in a UV spectrophotometer at 625 nm. The MIC was defined as the lowest concentration of EOLC capable of completely inhibiting visible bacterial growth. All assays were performed in triplicate, and the results were expressed as mean ± standard deviation.

### 2.5. Toxicity Bioassay Against Artemia salina

The test was performed according to Martins et al. [37], with modifications. In an aquarium, a saline solution prepared with 20 g of sea salt was added to 1.0 L of distilled water. The solution, with a pH between 8 and 9, was then exposed to the light of a 40 W lamp with aeration, and *A. salina* cysts were added for hatching. After 24 h, 10 nauplii were placed in test tubes containing EOLC samples dissolved in 1% DMSO. Starting from 20 mg of EOLC, concentrations between 150 and 1000 µg/mL were tested in triplicate. As a control test, DMSO and saline solutions prepared using a method similar to that for the samples were used. After the 24 h incubation period, the number of surviving nauplii in each bottle was counted and the Lethal Concentration 50 (LC_50_) was calculated using the OriginPro 2023b software.

### 2.6. Antioxidant Activity and Total Phenolic Compounds (TPC)

To determine the antioxidant activity (DPPH and TPC), an extract was prepared from the dilution of EOLC (1:10 *V*/*V* methanol). The determination of ABTS radical scavenging activity was performed directly from EOLC.

#### 2.6.1. DPPH Radical Scavenger Activity

The antioxidant activity of EOLC was evaluated by measuring the reduction of DPPH radicals, following the methodology described by Brand-Williams et al. [38] and Campos et al. [39]. For the assay, a 75 μL aliquot of the EOLC stock solution was added to 4 mL of the DPPH solution. The samples were then incubated in the dark for 30 min, and the absorbance of the solutions was measured at 515 nm using a spectrophotometer. The antioxidant activity was determined using a standard Trolox calibration curve (6-hydroxy-2,5,7,8-tetramethylchroman-2-carboxylic acid; Sigma-Aldrich). Results were expressed as micromoles of Trolox equivalents (μMol TE). The calibration curve was described by the equation y = −19.455x + 0.5098, where y represents the absorbance value and x the Trolox concentration expressed in μMol, with a coefficient of determination R^2^ = 0.9974.

#### 2.6.2. ABTS Radical Scavenging Activity

Antioxidant activity was determined according to the methodology described by Re et al. [40], with modifications. The ABTS^+•^ radical was generated through the reaction of 5 mL of ABTS solution (7 mM) with 88 μL of potassium persulfate solution (140 mM). The mixture was kept in the dark at 25 °C for 16 h. For the test, the activated ABTS^+•^ radical was diluted in ethanol to an absorbance of 0.70 at 734 nm. A 10 μL aliquot of the EOLC was added to 1 mL of the diluted ABTS^+•^ solution, and the absorbance (734 nm) was measured after 6 min. Antioxidant activity was calculated using a standard Trolox calibration curve, and the results were expressed as Trolox equivalent antioxidant capacity (TEAC) in mM. The calibration curve was described by the equation y = −0.005x + 0.9963, where y represents the absorbance value and x the Trolox concentration expressed in μMol, with a coefficient of determination R^2^ = 0.9913.

#### 2.6.3. Total Phenolic Compounds

Total phenolic compounds (TPC) were determined according to the methods described by Genovese & Lannes [41] and Campos et al. [39]. An aliquot of the previously prepared EOLC extract (200 μL) was mixed with Folin–Ciocalteu reagent (300 μL), 2 mL of a 15% sodium carbonate solution, and distilled water to complete the volume in a 5 mL volumetric flask. The solutions were centrifuged (5170× *g* for 10 min), incubated at room temperature in the dark (25 °C, 120 min), and the absorbance was measured at 798 nm. The result was obtained from the calibration curve with gallic acid and expressed as milligrams of gallic acid equivalent per gram of sample (mg GAE/g).

### 2.7. Determination of the Physicochemical Properties of EOLC

#### 2.7.1. Relative Density

A pycnometer with a capacity of 1 mL was used at an ambient temperature of 25 °C. The pycnometer was previously cleaned, dried, and then weighed empty on an analytical balance until a constant weight was obtained. Then, the pycnometer was filled with 1 mL of EO and closed, and the excess oil was wiped off. The pycnometer with EO was weighed, and the relative density was determined from the difference in mass, according to the following Equation (2) [42]:

(2) $$\rho \;(g/mL)=\frac{\left(Pycnometer\;weight+sample)-(Pycnometer\;weight\right)}{Essential\;oil\;volume}$$

#### 2.7.2. Refractive Index

To determine the refractive index of the essential oil, Pasteur glass pipettes were used to add two drops of the oil directly onto the Flint prism of the refractometer at 20 and 25 °C. For the refractive index measurements, an Abbe refractometer [42] was used.

#### 2.7.3. Viscosity

The kinematic viscosity of the essential oil was determined using a Cannon–Fenske type viscometer, SCHOTT brand, model AVS 350, according to the ASTM D445-17 method, based on the methodology described by Ruiz-Gonzalez et al. [43]. Capillary tube number 50 was used, with a constant k of 0.003899 mm^2^/s and a viscosity range between 0.8 and 3.2 mm^2^/s. Analyses were carried out at temperatures of 20 and 25 °C. For each temperature, average flow time values of the oil through the measuring bulb were recorded. Viscosity was calculated based on Equation (3).

(3) $$v=k.t_{o},$$

in which *v* is the kinematic viscosity in mm^2^/s; k is the constant for capillary no. 50 in mm^2^/s; and t_o_ is the average time that the oil passes through the measuring bulb in seconds (s).

#### 2.7.4. Ethanol Solubility

Solubility was determined in a 1.5 mL Eppendorf tube, with the addition of 1000 μL of 70% (*v*/*v*) ethanol and 20 μL of essential oil. The mixture was then homogenized in a vortex at 2800 rpm for 1 min, as described by Alarcón et al. [42]. 

### 2.8. Sensory Analysis of Lamb Burgers Incorporated with EOLC

The sensory analysis study was approved by the Research Ethics Committee involving human beings. Federal University of Roraima (protocol numbers: 23282819.9.0000.5302, on 17 January 2024), Boa Vista, State of Roraima, Brazil.

To evaluate the inclusion of essential oil extracted from cashew tree leaves in foods, two concentrations were tested based on previous studies [44,45].

A panel of 20 individuals (research team) evaluated the color, appearance, odor, flavor, and tenderness of lamb burger samples containing different concentrations of essential oil in their formulation 0.05% (*p*/*p*) and 0.1% (*p*/*p*). Some panelists had extensive experience in conducting sensory evaluations of different food products. Members who had no experience were trained in a training session on the use of the hedonic scale and the quality attributes that are used for sensory analysis according to Ghabraie et al. [46].

The burgers were individually packed with aluminum foil and cooked on a pre-heated grill at 200 °C until the internal temperature reached 72 °C. The samples were served separately and identified by 3 random digits.

The evaluation was carried out using a 9-point hedonic scale: (1 = extremely disliked; 9 = extremely liked), without the average level, according to Leite et al. [47].

### 2.9. Statistical Analysis

Statistical analysis was performed using SPSS software (version 23.0; IBM SPSS Statistics, SPSS Inc., Chicago, IL, USA) for Windows. Response variables were subjected to analysis of variance (ANOVA), and means were compared using Tukey’s test at a 5% significance level. Results are expressed as mean ± standard deviation (SD), based on triplicates for each experimental condition.

For the consumer sensory analysis, the experimental design considered treatment (essential oil concentration) as a fixed effect and the consumer as a random effect. The response variable was the sensory acceptability of the samples.

## 3. Results and Discussion

### 3.1. Yield of EOLC

The yield of essential oil obtained from frozen leaves was 0.02% ± 0.00, higher than that observed for fresh leaves, which presented a yield of 0.01% ± 0.00, with both results obtained from triplicate extractions. Previous studies report variable yields for the essential oil of *A. occidentale* leaves by hydrodistillation, with values lower than 0.1% [10,48], and others higher, between 0.1% and 0.3% [11,49]. Variations in the yield of essential oils can be explained by several factors, including the period of plant material collection, stage of plant maturation, drying method and agroecological conditions [50,51].

Furthermore, storage conditions can affect the yield of essential oils, as demonstrated by Mei et al. [52], when they evaluated the effect of cooling, freezing and drying on the essential oil yield of *Eucalyptus globulus* leaves. The authors obtained a higher oil yield in frozen samples, which was observed the same in our study. When analyzing images obtained by scanning electron microscopy (SEM), Pathan et al. [53] observed that after drying the leaves, the epidermal cells shrank and the stomata closed, while in the frozen leaves they formed irregularly shaped semi-crystalline parts. Freezing leaves can cause the formation of ice crystals that rupture the cell walls, facilitating the release of volatile compounds during hydrodistillation [52], which may explain the higher yield obtained for the essential oil extracted from frozen leaves.

A preliminary analysis of the chemical composition of the essential oils extracted from fresh and frozen leaves of the dwarf cashew tree was also conducted, and no significant differences were observed between them. These results reinforce the viability of this storage method, preserving the quality of the essential oil.

Thus, the use of frozen plant material not only improves the oil yield but also offers a practical solution for preserving leaves over extended periods, contributing to greater logistical flexibility for industrial applications and scientific research.

Even with the low yield of essential oil from *A. occidentale* leaves, small amounts were sufficient to allow the incorporation of the ingredient into food formulations, as validated in our sensory acceptability test, following the regulations and reinforcing that small additions of bioactive compounds may be sufficient to promote desirable effects in foods, replacing synthetic antioxidants that are related to diseases [44,54]. This consideration reinforces the exploratory nature of the present study and the potential application of the essential oil in low doses.

### 3.2. Chemical Composition of EOLC

Due to the higher yield of essential oil obtained from frozen leaves and the preservation of its constituents, this sample was selected for biological, physicochemical and acceptability analyses. In the EOLC sample, 30 chemical constituents were identified (Table 1).

**Figure 1:**
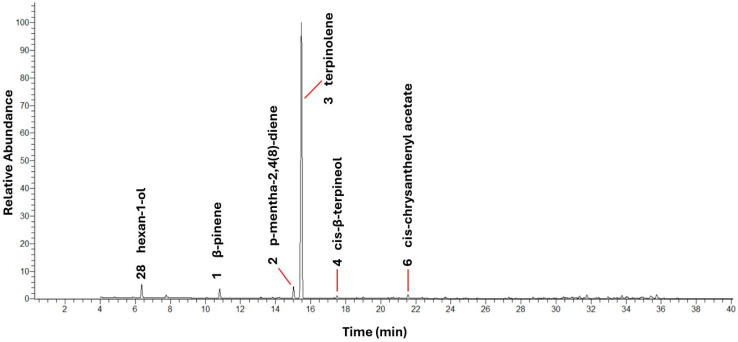
Representative GC-MS chromatogram of EO of the early dwarf cashew tree CCP 76.

The essential oil from cashew tree leaves is predominantly constituted of monoterpene hydrocarbons, which include β-pinene, p-mentha-2,4(8)-diene and, mainly, terpinolene. These three compounds represented 85.26% of the total composition, with terpinolene being the major component, with 80.21% (Figure 1). The high concentration of this compound was also observed by Sousa et al. [9], who identified terpinolene in high proportion in the volatile metabolomic profile of the leaves of the early dwarf cashew tree CCP 76. In comparison, in the essential oil from the leaves of *Astronium fraxinifolium* Schott ex Spreng, another species belonging to the Anacardiaceae family, terpinolene was identified as one of the main constituents, however, with a significantly lower concentration (15.2%) [49]. On the other hand, in cashew trees from other regions, such as the Nigerian cashew tree, limonene was the predominant compound, representing 85.9% of the essential oil in the leaves [48]. These contrasts highlight the influence of genotype and environmental conditions on the composition of essential oils.

Kossouoh et al. [10], when evaluating the chemical composition of the essential oil from the leaves of *A. occidentale*, identified the sesquiterpenes germacrene B, germacrene D, β-caryophyllene and δ-cadinene as the main constituents. Similarly, in the study conducted by Montanari et al. [49], the major compounds were (E)-caryophyllene (15.4%) and germacrene D (11.5%), although other sesquiterpenes, such as α-copaene, α-cadinene, bicyclogermacrene and germacrene B, were also identified in smaller proportions

The variation in the chemical profiles of essential oils extracted from the same species can be explained by factors such as geographic location, climate, soil composition, plant development stage, among others, which directly influence the biosynthesis of secondary metabolites [55,56,57]. These differences, in comparison with the results obtained in the present study, which showed the predominance of monoterpenes, especially terpinolene, highlight the diversity in the chemical composition of *A. occidentale* essential oils.

Terpinolene, identified as the main component of the essential oil in this study, is also found in the essential oils of various plant species, such as *Elaeoselinum asclepium* (L.) Bertol [58], *A. occidentale* [9], and *A. fraxinifolium* [49]. Several studies have reported that this monoterpene exhibits relevant biological activities, including anti-inflammatory, antioxidant, and anticancer properties, as well as a lack of genotoxic effects [59,60].

### 3.3. Antibacterial Activity

**Figure 2:**
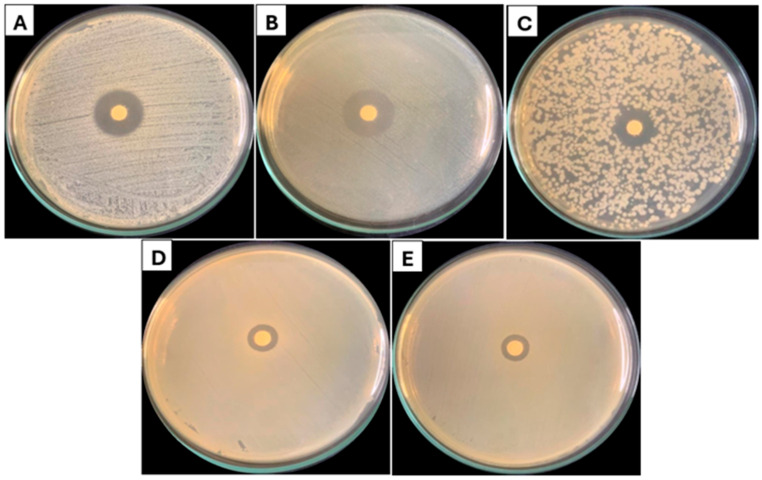
EOLC growth inhibition zone against: (**A**) *Staphylococcus aureus*, (**B**) *Listeria monocytogenes*, (**C**) *Bacilus cereus*, (**D**) *Salmonella enteritidis*, and (**E**) *Escherichia coli*.

The EOLC evaluated in this study demonstrated the ability to inhibit the growth of the tested bacteria, as evidenced by the presence of inhibition zones (Figure 2) and by the results obtained by broth microdilution. Significant differences (*p* < 0.05) were observed in the diameters of the inhibition zones and in the MIC values between Gram-positive and Gram-negative bacteria (Table 2).

Gram-positive bacteria showed broader zones of inhibition, indicating greater susceptibility to the essential oil. Considerably, the EOLC showed the formation of larger zones of inhibition against the bacteria *Listeria monocytogenes* (21.50 mm) and *Staphylococcus aureus* (18.66 mm), compared to the bacteria *Salmonella enteritidis* (11.66 mm) and *Escherichia coli* (11.33 mm), both Gram-negative species. In the present study, the Minimum Inhibitory Concentration (MIC) results reinforce the pattern of differential sensitivity observed between Gram-positive and Gram-negative bacteria. The EOLC showed lower MIC values for Gram-positive strains, such as *Staphylococcus aureus* (0.25 mg/mL) and *Bacillus cereus* (0.31 mg/mL), while higher values were found for Gram-negative strains like *Escherichia coli* (1.00 mg/mL) and *Salmonella enteritidis* (1.50 mg/mL) (Table 2). These findings are consistent with the general antimicrobial profile of essential oils.

Similarly, Montanari et al. [49], when evaluating the antimicrobial activity of essential oils extracted from the leaves of various Anacardiaceae species, including *A. occidentale*, reported a more pronounced effect against Gram-positive bacteria. Alia et al. [61] also demonstrated significant inhibitory activity of essential oils from the shoots of several *A. occidentale* clones, particularly against *S. aureus*. Consistent with these findings, Rocha et al. [62] observed reduced sensitivity of Gram-negative bacteria to essential oil extracted from the leaves and fruits of *Schinus molle* L., another species within the Anacardiaceae family.

Fancello et al. [63], when evaluating the antimicrobial activity of essential oil from *Citrus limon* var. *pompia* leaves against food associated microorganisms, observed that, as expected, *Salmonella enterica* and *E. coli* showed resistance to the essential oil. These findings support several studies demonstrating greater susceptibility of Gram-positive bacteria to citrus essential oils compared to Gram-negative bacteria [64,65].

This lower susceptibility of Gram-negative bacteria is associated with their complex cell wall structure, composed of multiple layers of peptidoglycan and an outer membrane, which hinders the diffusion and accumulation of lipophilic compounds, such as essential oils, within the bacterial cell [66]. As highlighted by Nazzaro et al. [67], the antimicrobial activity of essential oils is mainly attributed to their ability to alter membrane permeability, disrupt enzymatic systems, and interfere with nutrient uptake. However, the outer membrane of Gram-negative bacteria acts as an effective barrier to hydrophobic molecules, contributing to their higher resistance when compared to Gram-positive strains.

It is worth noting that Montanari et al. [49] reported that MIC values do not necessarily follow the same order as inhibition zone diameters observed in agar diffusion assays, due to the limited diffusion of hydrophobic compounds in solid media. This highlights the importance of combining both methodologies to obtain a more comprehensive understanding of antibacterial activity.

The results obtained in this study confirm the potential of EOLC as a natural antimicrobial agent, with greater efficacy against Gram-positive bacteria, suggesting its possible application as a preservative of plant origin in food products sensitive to microbial contamination.

### 3.4. Evaluation of EOLC Toxicity in Artemia salina

**Figure 3:**
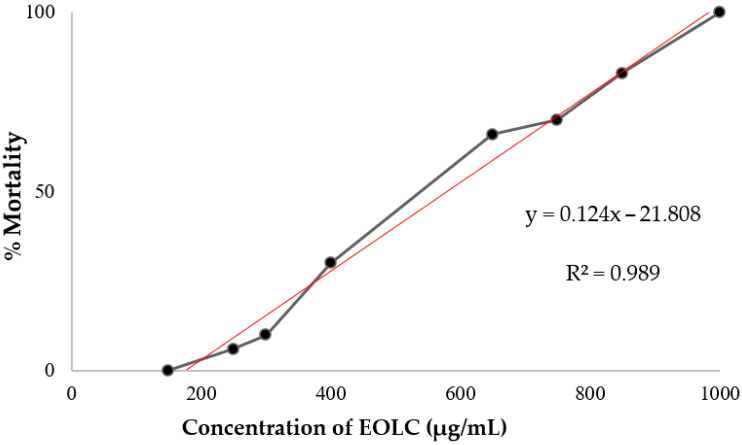
Mortality of *A. salina* exposed to different concentrations of EOLC. The linear regression line, in red, represents the data fit, indicating an increasing trend in mortality as a function of concentration.

Studies have classified organic and aqueous extracts into different degrees of toxicity. Based on the ranges in which extracts with an LC_50_ above 1000 μg/mL are considered non-toxic, an LC_50_ between 1000 and 500 μg/mL is classified as moderately toxic. If an LC_50_ is in the range of 100 to 500 μg/mL, the toxicity is considered low, and extracts with an LC_50_ lower than 100 μg/mL are classified as highly toxic [37,68]. The results obtained in this study showed that the EOLC presents moderate toxicity, with an LC_50_ equal to 579.09 ± 1.048 μg/mL (Figure 3).

This toxicity may be related to the high concentration of terpinolene, a monoterpene with recognized cytotoxic action, the effect of which varies according to the time of exposure and the concentration used [60]. Essential oils rich in this compound, such as those obtained from the leaves of *Myrciaria floribunda* (H. West ex Willd.) O. Berg and *Duguetia echinophora* R.E.Fr., showed moderate (LC_50_ = 82.96 μg/mL) and high (LC_50_ = 28.00 μg/mL) toxicity, respectively [69,70]. Furthermore, terpinolene has been identified as a potential antiproliferative agent in brain tumor cells [59].

The *A. salina* assay is widely recognized as a preliminary toxicological screening tool for bioactive compounds [37]. Studies demonstrate that plant extracts and essential oils with toxicity detected in the *A. salina* assay often exhibit desirable biological properties, such as antioxidant, antimicrobial, selective cytotoxic or antifungal action, reinforcing the use of this model as a preliminary screening for natural bioactive compounds [71].

In the EOLC evaluated in our study, the main component identified was terpinolene (85.26%), which is classified as GRAS (Generally Recognized as Safe) by FEMA (Flavor and Extract Manufacturers Association) for use as a food flavoring, being derived from natural processes of plant biosynthesis, and the natural occurrence of these compounds in essential oils of fruits, spices, vegetables, peels, roots and leaves reinforces their role as common components of the human diet Adams et al. [72].

The EOLC dosages used in the lamb burger formulations were defined experimentally based on sensory acceptability tests [46] and are within the concentration allowed for BHT in meat products according to Brazilian regulations. Furthermore, the concentrations used were below the LC_50_ determined in the *Artemia salina* toxicity bioassay (579 µg/mL), reinforcing an adequate safety margin.

Since the EOLC was incorporated into a solid food matrix, its bioavailability was likely further reduced compared to applications in aqueous systems. This is relevant, as the direct use of essential oils in foods has known limitations due to their low water solubility, high volatility, and reduced stability and bioavailability [73,74].

Despite these limitations, the concentrations tested in this study were sufficient to provide desirable sensory attributes, particularly in terms of odor and flavor, as observed in the acceptability tests. These findings reinforce the potential of the essential oil from *Anacardium occidentale* leaves (EOLC) as a safe and effective natural preservative, offering a viable and health-conscious alternative to synthetic antioxidants currently used in the country.

### 3.5. Antioxidant Activity

The antioxidant activity of EOLC analyzed using DPPH and ABTS radical scavenging assays and the content of phenolic compounds are shown in Table 3.

The antioxidant capacity of EOLC, as determined by the DPPH radical scavenging assay, was 1.96 µmol Trolox mL^−1^. Similar values have been reported for essential oils from Apiaceae family species widely used in food, such as *Cuminum cyminum* L. (cumin), *Foeniculum vulgare* Mill. (fennel), *Coriandrum sativum* L. (coriander), and *Daucus carota* L. (carrot), which showed DPPH activities of 2.74, 1.73, 1.18, and 0.26 µmol Trolox g^−1^, respectively [75]. This similarity reinforces that EOLC has an antioxidant potential comparable to that of species traditionally used as condiments and natural ingredients, which may support its future applications in the food industry.

The antioxidant activity of the EOLC was also evaluated using the ABTS^+•^ method, yielding a value of 1.41 mM. This result was higher than that reported by Ferreira et al. [76], who analyzed the essential oil extracted from the leaves of *Eugenia florida* DC. from different regions and found an average activity of 0.554 mM. Additionally, the essential oil extracted from the fruits of *Schinus molle* L., a species belonging to the same family as the cashew tree, showed an antioxidant activity of 4.7 mM by the ABTS method, a value considered effective in neutralizing free radicals, according to Eryigit et al. [77]. These results indicate that EOLC presents relevant antioxidant activity, with favorable performance compared to other species of medicinal interest [76]. Considering that the ABTS^+•^ method is sensitive to both hydrophilic and lipophilic compounds [40], the observed value may be related to the presence of monoterpenes with antioxidant potential, such as terpinolene [78], identified as the main constituent of EOLC.

The essential oil of the early dwarf cashew tree showed a total phenolic content of 0.38 mg GAE/g. The antioxidant activity of many plants is often associated with the presence of these compounds [79], as demonstrated by Guo et al. [80], who analyzed essential oils from *Thymus vulgaris* L. (red thyme), *Origanum vulgare* L. (oregano), and *Satureja hortensis* L. (summer savory), as well as oils extracted from *Syzygium aromaticum* L. (clove) and *Cinnamomum zeylanicum* Blume (cinnamon), all of which are rich in phenolic compounds such as carvacrol, thymol, and eugenol. The authors attributed the high antioxidant capacity of these oils to their elevated phenolic content.

In the EOLC, terpinolene stands out as the major compound, a monoterpene recognized for its antioxidant activity. Previous studies have demonstrated the effectiveness of terpinolene in scavenging free radicals, reinforcing its potential as a natural and safe antioxidant when used at low concentrations [59,60]. Furthermore, as reported by Lu et al. [78], terpinolene exhibited antioxidant activity comparable to that of the synthetic standard BHT (butylated hydroxytoluene), further supporting its applicability as a natural alternative in food and pharmaceutical formulations. The presence of a strongly activated methylene group in its structure may be responsible for this behavior [81].

The results presented here contribute to the understanding of the antioxidant potential of the essential oil from early dwarf cashew tree leaves and support its possible application as a functional ingredient. Studies such as that by Muanda et al. [82], which demonstrated significant antioxidant properties in the essential oil and extracts of *Stevia rebaudiana* Bertoni, highlight the relevance of exploring natural compounds with multiple functionalities, as observed in the EOLC.

Considering that EOLC showed antioxidant and antibacterial activities, it is pertinent to discuss a possible relationship between these biological properties. Although the mechanisms involved are distinct, it is possible that certain compounds present in the EOLC, such as terpinolene, the major constituent of the sample, may have multifunctional potential, contributing to both observed effects. Studies indicate that these functions may coexist in essential oils with specific chemical profiles [83,84]. This dual activity has been frequently reported in essential oils applied to foods, in which such properties contribute to the microbiological preservation and oxidative stability of products [85].

### 3.6. Assessment of the Physicochemical Characteristics of EOLC

The results of the physicochemical properties of the essential oil obtained from the leaves of the early dwarf cashew tree are presented in Table 4. The values of the physicochemical parameters indicate the characteristics of the essential oils, being fundamental for the evaluation of quality [86].

The density and refractive index of substances are directly related to their chemical composition [87]. In this study, the EOLC density value was 0.840 mg/mL at 25 °C, a value comparable to the essential oil of other species, such as *Pistacia vera* L., also from the cashew tree family, with a density of 0.89 g/cm^3^ [88], *R. officinalis* essential oil with a density of 0.907 g/cm^3^, *Cinnamomum verum* J.Presl essential oil with a density of 1024 g/cm^3^ and *Origanum vulgare* L. essential oil with a density of 0.946 g/cm^3^ [89].

The refractive index value of EOLC was 1.49 at 20 °C and 1.50 at 25 °C. The refractive index of pure essential oils generally ranges between 1.450 and 1.590, and they are characteristic of each oil [90]. Therefore, the values presented in this study were in accordance with the quality range. Furthermore, the EOLC refractive index values of are close to those reported for other essential oils in the literature, such as *Lavandula angustifolia* Mill. (1.4565), *Salvia officinalis* L. (1.4712), *Ocimum basilicum* L. (1.4692), *Brassica napus* L. (1.4690) [91] and *Pistacia vera* L. (1.4693–1.4700) [88].

The viscosity of the EOLC decreased as the temperature increased, in accordance with what is expected for liquid substances [92]. Furthermore, the values obtained are within the viscosity variation range (0.8–3.2 mm^2^/s) for capillary number 50, with a constant equal to 0.003899 mm^2^/s^2^.

For solubility in 70% ethanol, the results demonstrated that the EOLC dissolved completely. For the solubility in ethanol of essential oils extracted from fruits, leaves and flowers of *Eucalyptus cinerea* F. Muell. ex Benth., Silva et al. [93] indicated that an amount greater than one part of 70% ethanol was necessary for the oil to become miscible. Essential oils of peppermint, cinnamon and thyme are mentioned by Stevanovic et al. [73] as being soluble in 70% alcohol, which was also observed in our study.

The results found for density, refractive index, viscosity and solubility of the essential oil from frozen cashew tree leaves exhibited the expected standards and are in accordance with the values found in the literature.

### 3.7. Sensory Properties of EOLC in Hamburger

Consumers were not able to distinguish (*p* > 0.05) differences in color, appearance, and tenderness among the hamburgers evaluated (Table 5).

For the attributes of odor and flavor, significant differences were observed between treatments (*p* < 0.05). Odor received the highest score for the 0.05% essential oil treatment, compared to the control (CON) and the 0.1% essential oil treatment, while the BHT treatment showed an intermediate value.

Regarding flavor, the highest scores (*p* < 0.05) were observed for the 0.05% essential oil and BHT treatments, while the control and 0.1% treatments received the lowest scores.

Considering the results obtained for odor and flavor, it is possible to suggest that the bioactive compounds present in EOLC have resisted heating, contributing to residual effects in the final product. During preparation, the hamburgers were baked at 200 °C until reaching an internal temperature of 72 °C, a condition that may favor the degradation of volatile compounds. However, evidence in scientific literature indicates that some essential oils can maintain significant antioxidant activity even after exposure to high temperatures, without substantial changes in chemical composition [94]. In addition, the hamburgers treated with EOLC presented relevant sensory differences, such as flavor and odor, as demonstrated in the acceptability tests (Table 5), which may suggest the maintenance of the content of active compounds after the thermal process. These findings reinforce the potential of EOLC as a natural preservative in meat products subjected to thermal processing.

Boskovic et al. [95] when evaluating the addition of thyme essential oil to pork found that sensory acceptance was greater in treatments with the addition of the lowest concentration of essential oil, a result similar to that found by Lages et al. [96]. in the sensory evaluation of meat sausage using thyme essential oil (*Thymus vulgaris* L.) and the same verified in our study.

## 4. Conclusions

The extraction of essential oil from frozen dwarf cashew tree leaves proved to be a viable strategy, with a higher yield than that of fresh leaves and the advantage of allowing the storage of plant material for long periods without compromising oil production. The EOLC showed antioxidant properties and antibacterial effects, especially against Gram-positive bacteria, in addition to physicochemical characteristics compatible with the standards expected for essential oils. The low toxicity observed, combined with antioxidants and antibacterial efficacy, confirms the potential of EOLC as a natural source of bioactive compounds for application in different types of products.

The concentration of 0.05% EOLC was the best accepted in sensory tests, indicating its viability as a natural alternative for application in meat products. Thus, the essential oil from A. occidentale leaves appears as a promising source of bioactive compounds, with prospects for use in different food matrices. Future studies should evaluate its technological efficacy and safety under real processing and storage conditions, expanding its use in food preservation.

## Figures and Tables

**Table 1:** Chemical composition of the essential oil from leaves of the early dwarf cashew tree (CCP 76).

No.	Constituents	RI ^1^	RI ^2^	EOLC (%)	Molecular Formula
Monoterpenes hydrocarbons				85.26	
1	*β*-Pinene	972	974	2.29	C_10_H_16_
2	*p*-Mentha-2,4(8)-diene	1081	1085	2.76	C_10_H_16_
3	Terpinolene	1093	1086	80.21	C_10_H_16_
Oxygenated monoterpenes				2.16	
4	*β*-Terpineol	1148	1140	0.63	C_10_H_18_O
5	*trans*-Sabinene hydrate acetate	1248	1253	0.22	C_12_H_20_O_2_
6	cis-Chrysanthenyl acetate	1262	1261	0.92	C_12_H_18_O_2_
7	*p*-Mentha-1,4-dien-7-ol	1325	1325	0.39	C_10_H_16_O
Sesquiterpenes hydrocarbons				2.21	
8	*β*-Copaene	1437	1430	0.3	C_15_H_24_
9	Germacrene D	1482	1480	0.38	C_15_H_24_
10	*α*-Muurolene	1501	1500	0.18	C_15_H_24_
11	*E*-*γ*-Bisabolene	1520	1528	0.13	C_15_H_24_
12	*α*-Cadinene	1541	1537	0.33	C_15_H_24_
13	Selina-3,7(11)-diene	1547	1545	0.25	C_15_H_24_
14	Germacrene B	1558	1559	0.64	C_15_H_24_
Oxygenated sesquiterpenes				6.71	
15	Palustrol	1566	1567	0.16	C_15_H_26_O
16	Spathulenol	1572	1577	0.65	C_15_H_24_O
17	Caryophyllene oxide	1581	1582	0.26	C_15_H_24_O
18	Globulol	1586	1590	0.84	C_15_H_26_O
19	Ledol	1605	1602	0.24	C_15_H_26_O
20	Di-*epi*-1,10-Cubenol	1610	1618	0.22	C_15_H_26_O
21	1-*epi*-Cubenol	1629	1627	0.43	C_15_H_26_O
22	*epi*-*α*-Cadinol	1644	1638	0.17	C_15_H_26_O
23	*α*-Cadinol	1657	1652	0.74	C_15_H_26_O
24	Intermedeol	1666	1665	0.17	C_15_H_26_O
25	Eudesm-7(11)-en-4-ol	1699	1700	0.79	C_15_H_26_O
26	(2*E*,6*Z*)-Farnesol	1717	1714	1.18	C_15_H_26_O
27	Guaiol acetate	1729	1725	0.86	C_17_H_28_O_2_
	Others			3.66	
28	Hexan-1-ol	851	863	3.1	C_6_H_14_O
29	Hexenyl-3-methyl butanoate	1238	1232	0.28	C_11_H_20_O_2_
30	cis-3-Hexenyl valerate	1285	1279	0.28	C_11_H_20_O_2_
	Total			100	

^1^ Calculated retention index in relation to the series of homologous n-alkanes (C8–C20) in the TR-5 column, ^2^ Literature retention index [33], NIST 20.

**Table 2:** EOLC growth inhibition zones and antibiotic growth inhibition zones.

Tested Bacteria	IZ *	Chloramphenicol (IZ *)	MIC (mg/mL)
*Staphylococcus aureus*	18.66 ^a^ ± 0.57	29.01 ^c^ ± 0.01	0.24 ^d^ ± 0.01
*Listeria monocytogenes*	21.50 ^a^ ± 2.29	38.01 ^a^ ± 0.01	0.56 ^c^ ± 0.05
*Bacillus cereus*	15.33 ^b^ ± 0.28	29.01 ^c^ ± 0.01	0.31 ^d^ ± 0.01
*Salmonella enteritidis*	11.66 ^c^ ± 0.57	34.01 ^b^ ± 0.01	1.00 ^b^ ± 0.10
*Escherichia coli*	11.33 ^c^ ± 0.57	29.01 ^c^ ± 0.01	1.52 ^a^ ± 0.04

* IZ: diameter of the growth inhibition zone. Different lowercase letters in the same column are significantly different.

**Table 3:** Antioxidant activity and total phenolic compounds of the EOLC of the early dwarf cashew tree.

Antioxidant Activity		Total Phenolic Compounds (mg EAG/g)
DPPH (µmol Trolox mL^−1^)	ABTS (mM TE)	
1.96 ±1.07	1.41 ± 0.04	0.38 ± 0.58

**Table 4:** Physicochemical parameters of the essential oil of the early dwarf cashew tree.

Parameter	EOLC
Density (g/mL)	0.84 ± 0.01
Refractive index 20 °C	1.49 ± 0.01
Refractive index 25 °C	1.50 ± 0.01
Viscosity (mm^2^/s) 20 °C	1.83 ± 0.10
Viscosity (mm^2^/s) 25 °C	1.67 ± 0.01
Solubility	Positive

**Table 5:** Consumer acceptability of lamb burgers treated with BHT and cashew tree leaf essential oil.

Treatments				
	CON ^1^	BHT ^2^	EO-0.05% ^3^	EO-0.1% ^4^
Color	5.45 ± 2.08	6.65 ± 1.66	6.75 ± 1.88	5.80 ± 2.09
Appearance	5.55 ± 2.46	6.65 ± 1.59	6.65 ± 2.00	6.20 ± 2.11
Odor	4.95 ^b^ ± 2.48	5.95 ^ab^ ± 2.25	7.10 ^a^ ± 1.58	4.95 ^b^ ± 2.43
Flavor	4.05 ^b^ ± 2.54	7.05 ^a^ ± 1.57	7.20 ^a^ ± 1.36	4.20 ^b^ ± 2.39
Tenderness	6.70 ± 1.94	7.20 ± 1.64	7.55 ± 1.53	6.90 ± 1.94

Means of treatments with different lower-case letters in the same line are significantly different (*p* < 0.05). ^1^ CON—lamb burgers, without antioxidants; ^2^ BHT—lamb burgers containing hydroxytolueneobutylated; ^3^ EO-0.05%—lamb burgers with 0.05% of cashew tree essential oil; ^4^ EO-0.1%—lamb burgers with 0.1% of cashew tree essential oil.

## Data Availability

The original contributions presented in this study are included in the article. Further inquiries can be directed to the corresponding author.
